# Consumption of a Coffee Rich in Phenolic Compounds May Improve the Body Composition of People with Overweight or Obesity: Preliminary Insights from a Randomized, Controlled and Blind Crossover Study

**DOI:** 10.3390/nu16172848

**Published:** 2024-08-26

**Authors:** Álvaro Fernández-Cardero, José Luis Sierra-Cinos, Laura Bravo, Beatriz Sarriá

**Affiliations:** 1 Department of Metabolism and Nutrition, Institute of Food Science, Technology and Nutrition (ICTAN), Spanish National Research Council (CSIC), C/Jose Antonio Novais 6, 28040 Madrid, Spain; alvafe22@ucm.es (Á.F.-C.); lbravo@ictan.csic.es (L.B.); 2 Department of Health Science, School of Health Science, Universidad International Isabel I de Burgos (Ui1), C. de Fernán González, 76, 09003 Burgos, Spain; joseluis.sierra@ui1.es; 3 Department of Nutrition and Food Science I, School of Pharmacy, Universidad Complutense de Madrid, Ciudad Universitaria s/n, 28040 Madrid, Spain

**Keywords:** coffee, polyphenols, chlorogenic acid, caffeine, obesity, overweight, metabolic syndrome, body composition

## Abstract

This study analyzes the effects on body composition and variables related to metabolic syndrome of two coffees with different degree of roasting and phenolic content. Sixty participants with body mass index between 25 and 35 kg/m^2^ and a median age of 51.0 years (Interquartile range 46.3–56) were recruited. The study was a controlled, randomized, single-blind crossover trial consisting in drinking three cups/day of roasted coffee (RC) or lightly roasted coffee (LRC) during 12 weeks with 2-week wash-out stages before each coffee intervention. LRC contained ≈400 mg of hydroxycinnamic acids and ≈130 mg of caffeine per 200 mL/cup while RC contained ≈150 mg of hydroxycinnamic acids and ≈70 mg of caffeine per 200 mL/cup. Along the study, in each of the six visits, blood pressure, body composition by bioimpedance, anthropometric measurements, and blood biochemistry were analyzed. The mean differences and *p* values were calculated using a linear mixed model (JASP.v.0.18.0.3). A total of 38 participants completed the study. After the consumption of both coffees, fat mass and body fat percentage (LRC: −1.4%, *p* < 0.001; RC: −1.0%, *p* = 0.005) were reduced, whereas muscle mass and muscle mass percentage slightly increased (LRC: 0.8%, *p* < 0.001; RC: 0.7%, *p* = 0.002). The decrease in fat percentage was greater with LRC compared to RC (−0.8%; *p* = 0.029). There were no significant changes in metabolic syndrome variables or in body weight. In conclusion, LRC was slightly superior at inducing changes in body composition.

## 1. Introduction

The prevalence of overweight and obesity is increasing at alarming rates. According to a report from the World Health Organization published in 2022, 61.6% of the Spanish population presents overweight or obesity [1]. Obesity is considered a multifactorial chronic condition that is associated with a higher risk of suffering from other non-communicable diseases such as type 2 diabetes mellitus, ischemic heart disease or different types of cancer [2]. Furthermore, abdominal obesity is one of the components of the metabolic syndrome, along with insulin resistance, dyslipidemia, and high blood pressure [3]. The prevalence of the metabolic syndrome in the Spanish population is around 31% [4].

One of the main causes of obesity is having a positive energy balance for a prolonged period [5]. However, a multitude of factors can influence the development of this condition, such as genetic or chronobiological factors and even the microbiota [5]. Therefore, obesity must be treated with a multifactorial approach aimed at promoting fat mass loss. Although there are effective pharmacological and surgical treatments, the first-choice therapy is intervening in lifestyle, promoting healthy eating habits and sufficient physical activity that results in an energy deficit [6]. However, adherence to these interventions is usually low, which translates into a high failure rate in people with overweight or obesity [6].

Therefore, it is necessary to investigate new approaches that can reduce fat mass with higher long-term adherence, and that act synergistically with changes in lifestyle, also with less side effects compared to pharmacological or surgical treatments [6]. Increasing the consumption of foods rich in bioactive compounds, such as polyphenols, could be such a useful strategy, since previous studies demonstrate that these compounds exert beneficial cardiometabolic effects and can protect against obesity [6].

It should be noted that coffee is the main dietary source of polyphenols, specifically phenolic acids, in Europe [7]. The chemical composition of coffee is complex, containing over 1000 different phytochemicals along with macro- and micronutrients [8]. However, its composition depends on the coffee variety, its quality, and the degree of roasting among other factors. Thus, the caffeine content in *Coffea arabica* is approximately 1.5% of the weight of the bean, while in *Coffea robusta*, it is close to 2.7% [8]. Importantly, the content of phenolic compounds and the antioxidant capacity of coffee can be drastically reduced by roasting [9,10].

Among the major polyphenols in coffee, hydroxycinnamic acids stand out, as well as both monocaffeoylquinic and dicaffeoylquinic acids, including chlorogenic acid (5-caffeoylquinic acid), with reported health beneficial effects [11]. Coffee consumption represents 78.2% of total 5-Caffeoylquinic acids intake, 97.5% of total 4-Caffeoylquinic acid and 92.4% of 3-Caffeoylquinic acid in Europeans’ diet, while other minor sources of these hydroxycinnamic acids are potatoes, apples, pears, stone fruits, and tea [7].

These compounds have been shown to have a lipid-lowering effect, improving carbohydrate metabolism, reducing fat mass and, due to its antioxidant and anti-inflammatory capacities, providing additional benefits in people with metabolic syndrome [12]. Other bioactive substances in coffee, such as caffeine (1,3,7-trimethylxanthine) or trigonelline, may also have beneficial health properties, exerting synergistic effects with hydroxycinnamic acids and the other bioactive phytochemicals present in this beverage [8,11]. Coffee is also the main source of caffeine among people older than 18 years, whilst other sources include tea, energy drinks or soft drinks, the latter being the most consumed by children and adolescents [13].

According to the European Food Safety Authority (EFSA) Panel on Dietetic Products, Nutrition and Allergies (NDA) caffeine consumption is safe in doses up to 5.7 mg per kg of body weight (approximately 400 mg per day for a 70 kg person) [14]. Furthermore, the European Society of Cardiology, in its 2018 guidelines for the management of arterial hypertension, stated that coffee may have long-term cardiovascular benefits based on data from a meta-analysis of prospective cohort studies, which included over 1 million patients and 36,352 cardiovascular events [15,16]. In another meta-analysis, coffee consumption has been associated with a reduced hypertension incidence [17]. In addition, moderate caffeine consumption has been associated with a reduced risk of cardiovascular and all-cause mortality in adults and in older patients with hypertension [18,19].

It is important to highlight that polyphenols are sensitive to high temperatures, and that chlorogenic acid content can be reduced by between 40% and 80% in a coffee with a high degree of roasting [9]. Consequently, the interest arises in developing a coffee rich in polyphenols, obtained with a low degree of roasting, that is organoleptically pleasant, and that can be used as a strategy to increase the intake of hydroxycinnamic acids in a simple and realistic way, i.e., by replacing a regular coffee with one with a higher polyphenolic content. According to the scientific literature, a moderate consumption of three to four cups of coffee a day could provide health benefits for most people [8]. Other intervention studies have shown that the consumption of a roasted and green coffee blend rich in polyphenols could reduce the percentage of body fat and blood pressure, improving fasting blood glucose and triglycerides, thus reducing the risk of suffering metabolic syndrome [20,21,22].

For all these reasons, the hypothesis of the present study is that the regular consumption of a lightly roasted coffee rich in phenolic compounds could reduce total weight, fat mass, and improve some cardiometabolic parameters related to metabolic syndrome in subjects with overweight or obesity, compared to a commercial coffee with a higher degree of roasting and lower polyphenol content. Therefore, the main objectives of this work were to study people with overweight or obesity in terms of (1) changes in weight and body composition induced by both types of coffee, and (2) to analyze the changes in the variables related to metabolic syndrome after consumption of both coffees.

## 2. Materials and Methods

### 2.1. Study Design 

A crossover, randomized, controlled, and single-blind intervention study was carried out. *Coffea arabica* (L.) beans of superior quality from the same batch, from Colombia, were roasted to two different degrees: the control coffee was roasted as usual for commercial uses (roasted coffee, RC), whilst the experimental lightly roasted coffee (LRC) was roasted for a shorter time in order to obtain coffee beans with a higher polyphenol content yet ensuring their organoleptic properties. Both types of coffees were supplied by Supracafé S.A. (Madrid, Spain), roasting the coffee in the days before the participant’s visits to ensure the quality of the products.

**Figure 1:**
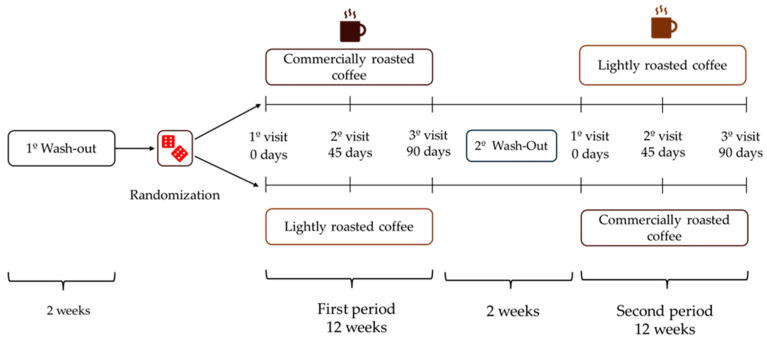
Design of the GREENCOF study, which is a randomized, controlled, single-blind crossover intervention.

After a 2-week wash-out period, volunteers were randomized to start with LRC or RC for 12 weeks (approximately 90 days), and then changed to the other coffee after a second 2-week wash-out period at the end of the initial coffee stage (Figure 1). The total duration of the study was seven months. Participants had to attend the Human Nutrition Unit (HNU) of the Institute of Food Science, Technology and Nutrition (ICTAN) 6 times in a fasting condition to carry out the determinations described below; the control visits were programmed at the beginning and end of each stage, and an intermediate visit. During the initial visit, information was obtained about the personal clinical history, medication, and sociodemographic data of the volunteers. 

During the coffee stages, volunteers consumed three cups of coffee (200 mL/cup) per day, prepared by following the instructions offered by the research team. In the initial and intermediate visits of each stage, participants were provided with the corresponding coffee enough until the next visit. The coffees were kept in opaque black bags, sealed and without identifying labels (blind study for volunteers). They were also provided with a French press coffee maker and a standardized scoop so that everyone could prepare the coffee under the same conditions. Commercial roasted coffee (RC) was prepared by adding 2 scoops (7 g) to a French press coffee maker, while lightly roasted coffee (LRC) was prepared with 4 scoops (14 g). Next, 200 mL of boiling water was added in both RC and LRC, stirred for a few seconds and allowed to infuse for 5 min before lowering the plunger and serving the coffee, which was consumed without milk, vegetable drinks or sugar, although artificial sweeteners were allowed. Coffee intake had to be separated from the main meals by at least 1.5 h and its consumption had to be spread throughout the day. Additionally, no other coffee, herbal infusions, cocoa derivatives, or other foods or supplements rich in caffeine or polyphenols could be consumed during the study. During the wash-out periods, participants could drink certain infusions such as black tea, chicory, malt, cocoa, or cereal drink, always with milk to promote the interaction of polyphenols with milk protein and thus reduce phenol bioavailability.

To ensure the correct coffee preparation, volunteers collected a 5 mL sample of the coffee freshly made in a 15 mL tube on each visit. These samples were analyzed by high-performance liquid chromatography with an online diode detector (HPLC-DAD), using calibration curves for chlorogenic acid and caffeine standards. If the phenolic content deviated by 10% from the reference value, participants were notified and reminded the correct way to prepare the coffee. In this manner, the total content of polyphenols and caffeine consumed by the participants would not exceed what was established. The content of chlorogenic acid and its derivatives (total hydroxycinnamic acids) in RC measured by HPLC-DAD was ≈150 mg per 200 mL, while in LRC it was ≈400 mg per 200 mL. In total, participants ingested approximately 1200 mg/day of polyphenols in the LRC stage, while in the CT stage they ingested 450 mg/day. The caffeine content was 130 mg/200 mL in LRC and 70 mg/200 mL in RC.

This study is registered in ClinicalTrials (NCT06204445). In addition, approval was obtained from the Clinical Research Ethics Committee of the Puerta de Hierro University Hospital (Report Code: PI 200/21) and the Bioethics Subcommittee of Spanish National Research Council (Report Code: 123/2022). The research protocols met the standards of the 1975 Declaration of Helsinki for studies in humans. To participate in the study, volunteers had to sign the informed consent before the intervention began. Participants were pseudonymized to hide their personal data, using random three-digit numerical codes. The results of this work have been reported as indicated in the extension for randomized crossover studies of the CONSORT 2010 (Consolidated Standards of Reporting Randomized Crossover Trials) guidelines, as can be seen in Supplementary Table S1 [23].

### 2.2. Inclusion/Exclusion Criteria, Recruitment and Randomization

Participants had to meet the following inclusion criteria:

(1) Body mass index (BMI) between 25.0 and 35.0 kg/m^2^.
(2) Age between 18 and 65 years old.

The exclusion criteria were as follows:

(1) People who followed a vegetarian or vegan diet.
(2) Smokers.
(3) Pregnant or breastfeeding women.
(4) People who took supplements that could interfere with the results of the study.
(5) People who had taken antibiotics two months before to the start of the study.
(6) People who have made recent changes in their eating habits and physical activity or who were planning to do so.

Furthermore, given that it is common for people with overweight/obesity to have other associated pathologies such as hypertension, dyslipidemia, or diabetes and, and are likely to be on pharmacological treatments, each case was analyzed to consider to what extent the treatment could interfere with the study. In general terms, people taking medication were allowed to participate as long as their conditions were well controlled and stable, and there had been no changes in the treatment drug or dose for at least six months before starting the intervention. Additionally, they were asked to communicate if there was any medical change during the study. 

The participants were recruited between October 2022 and May 2023. The study was advertised through the website of the GREENCOF project (www.proyectogreencof.es accessed on 1 June 2024), sending emails to workers of CSIC, and the Complutense University of Madrid as well as members of the Spanish Society for the Study of Obesity (SEEDO). In addition, posters and leaflets were distributed around the university campuses, pharmacies, cultural centers and areas surrounding the ICTAN. Other forms of recruitment included informative talks and advertisement of the project through social networks. The ICTAN’s volunteer database was used to contact potentially interested people and the Spanish Society of Clinical, Family and Community Pharmacy (SEFAC) also collaborated in the recruitment.

Volunteers were randomly assigned to the LRC-RC or RC-LRC sequence in a 1:2 ratio due to problems with the experimental coffee supply. Randomization was carried out by one of the main researchers (L.B.) by generating random numbers in Excel (Microsoft Excel, Professional Plus 2019, version 1808).

### 2.3. Diet Control, Measurement of Energy Expenditure, and Physical Activity

Volunteers were instructed to maintain their eating habits or physical activity during the study. Diet was monitored by interviewing participants by phone and recording three 24 h recalls (two on working days and one on a weekend day or holiday) on the days before each visit. Subsequently, the diet was analyzed with the DIAL program (Version 3.15.3; Department of Nutrition and Food Science of Complutense University of Madrid and Alceingeniería, S.A, Madrid, Spain).

Physical activity was monitored using accelerometers (ActiGraph wGT3X-BT; CamNtech Ltd., Pensacola, FL, USA). The participants had to wear the accelerometers for 7 consecutive days, removing them only to shower or to swim, recording in this case the swimming time. Using the ActiLife 6 software (ActiGraph version 6.13.5.) high-resolution raw acceleration data were transformed into other variables such as the average physical activity expenditure per day (kcal/day) and per hour (kcal/hour), the average step count per day, or the Metabolic Equivalent of a Task (MET), considering that one MET was equal to 3.5 mL of oxygen per kilogram of body weight per minute (mL/kg/min). 

Resting energy expenditure (REE) was estimated by indirect calorimetry (FITMATE Pro: portable calorimeter, version 4.10, Cosmed, Bicester, UK).

### 2.4. Analysis of Blood Samples and Blood Pressure

In each visit, participants attended the HNU after an overnight fast. Blood was extracted by a nurse, centrifuged to separate plasma or serum, and then analyzed by an external laboratory following the methods recommended by the Spanish Society of Clinical Biochemistry and Molecular Pathology (SEQC) using a Roche Cobas Integra 400 plus analyzer (Roche Diagnostics, Mannheim, Germany). For the present work, biochemical variables related to metabolic syndrome were analyzed, such as fasting blood glucose (FBG), triglycerides (TG), and high-density lipoprotein cholesterol (HDL).

Systolic and diastolic blood pressure was measured at each visit using an automatic sphygmomanometer (OMRON, Healthcare M3; Model: HEM-7131-E; Shanghai, China), following the protocol of the Spanish Society of Internal Medicine. Blood pressure was taken in both arms using a cuff of appropriate size, selecting the arm that showed a higher systolic value. Then two more measurements were subsequently made on the selected arm.

### 2.5. Anthropometric and Body Composition Measurements

In each control visit, anthropometric and body composition measurements were performed by a trained anthropometrist with ISAK 2 (International Society for the Advancement of Kinanthropometry) certification following the recommendations of this organization. Height was measured with a precision stadiometer (Holtain Ltd., Crymych, Pebs, Wales, UK) and weight with an OMRON scale (Body Composition Monitor BF511, OMRON, Hoofddorp, The Netherlands). Body mass index (BMI) was calculated as the ratio of body weight in kilograms to height in squared meters (kg/m^2^). Waist circumference was taken using a Cescorf anthropometric tape (Porto Alegre, Brazil). Body composition was determined with multifrequency tetrapolar bioimpedance (InBody S10, Co., Ltd., Cheonan-si, Republic of Korea), obtaining fat mass (kg), body fat percentage, skeletal muscle mass (kg), percentage of skeletal muscle mass and visceral fat area (cm^2^).

### 2.6. Sample Size Calculation

Sample size was calculated using the G Power 3.1.9.7 program. Changes in body weight were taken as the main variable, with an average difference of 2.5 kg and a standard deviation of 6.5 between the beginning and the end of the intervention [24]. The confidence level was set at 95% for a type I error (two-tailed Zα-score: 1.96) and the statistical power at 80% for a type II error (β = 0.2), obtaining a minimum sample size of 38 volunteers.

### 2.7. Statistical Analysis

Statistical analysis was performed using JASP (version 0.18.3.0). Continuous quantitative data were analyzed for normality using boxplots, quantile–quantile plots (Q-Q plots) and with the Shapiro–Wilk test. In addition, Levene’s test was used to check if there was equality of variances (homoscedasticity).

Regarding the initial sociodemographic characteristics of the study population, the categorical variables were expressed with absolute frequencies (*n*) and relative frequencies in percentage, while the continuous quantitative data that met the assumption of normality were expressed using the mean and standard deviation. Continuous quantitative data that did not meet normality were expressed with the median and interquartile range. The continuous variables of people assigned to follow LRC-RC or RC-LRC were compared using the student’s *t*-test for independent groups or the Mann–Whitney *U* test. Categorical variables were analyzed using the Chi-Square test (χ^2^) with continuity correction.

Finally, two linear mixed models were generated. First, the dietary variables (energy, macronutrients, fiber and alcohol) were analyzed at the beginning and at the end of each period, introducing sex, type of coffee, and time as fixed effect variables. The second model was generated by introducing the variables sex, type of coffee, time, the sequence in which the participants were randomized to drink the coffees and the interaction between type of coffee and time as fixed effects variables, while the random effects grouping factor was the code of each participant. This model was used to analyze the primary objectives (body composition, body weight, and variables related to metabolic syndrome). Holm’s method was used as a correction for multiple *post hoc* contrasts. The model also verified that there was no carry-over effect between interventions. With these models, the estimated marginal means, the standard error, and the pre-post intervention mean differences with the 95% confidence intervals (CI) were obtained. *p* values less than 0.05 were considered significant.

## 3. Results

As is shown in the flow chart based on the CONSORT 2010 guidelines for crossover intervention studies [23], 38 participants completed the study (Figure S1; Supplementary Material). Initially, 60 participants who met the inclusion criteria were recruited, but there was a high dropout rate due to the duration and logistics of the intervention trial. However, the minimum sample size required to carry out a preliminary analysis of the results was reached (*n* = 38).

Table 1 shows the sociodemographic data of the participants, collected at the first visit. Global and segmented data are shown based on the sequence to which the participants were randomized (LRC-RC or RC-LRC), following the CONSORT 2010 guidelines [23]. No significant differences were observed between participants who began with the LRC-RC sequence and those who started with the RC-LRC sequence in terms of sex distribution, educational level, age, BMI, or income level (Table 1). 

Table 2 shows the results of energy expenditure due to physical activity in kilocalories per day, the metabolic equivalents of tasks (METs), the average hourly kcals, the average steps per day, as well as the resting energy expenditure (REE) measured by indirect calorimetry.

No significant differences were found between the beginning and the end of each stage in the intake of macronutrients, total energy, dietary fiber or alcohol (Table 3). The percentage of energy from macronutrients, alcohol, and fiber also remained constant between the beginning and the end of each stage (Table 4). All this confirms that, as instructed, volunteers did not introduce changes in their diet during the intervention.

Table 5 shows the changes throughout the two coffee intervention stages in body composition, body weight and in the five diagnostic criteria of metabolic syndrome (waist circumference, fasting blood glucose, HDL cholesterol, triglycerides, and systolic and diastolic blood pressure). No carry-over effect was observed between either stage of intervention when comparing the baseline values of each period. The order in which participants consumed the two coffees (LRC-RC or RC-LRC sequence) neither had significant effects on the results.

No significant changes in body weight were observed in either period (Table 5). However, after examining the changes in the fat compartment measured with bioimpedance, a significant reduction in fat weight and fat percentage was detected at the end of both intervention stages, although the changes were greater with LRC. Even though the fat percentage values were similar at the beginning of both periods (35.3% at the beginning of the intervention with LRC compared to 35.7% with RC; *p* = 0.236), the difference between the end of the stage in which they consumed LRC compared to the end of the stage with RC was statistically significant (33.9% in LRC vs. 34.7%; mean difference = −0.8%, 95% CI, −1.4, −0.2; *p* = 0.029), pointing to the potential of LRC reducing body fat percentage. Regarding fat mass, although the loss after LRC was slightly higher compared to RC, the difference was not statistically significant when comparing the end of both interventions (28.9 kg in LRC vs. 29.5 kg in RC; mean difference = −0.7 kg, 95% CI, −1.3, −0.05; *p* = 0.069).

**Figure 2:**
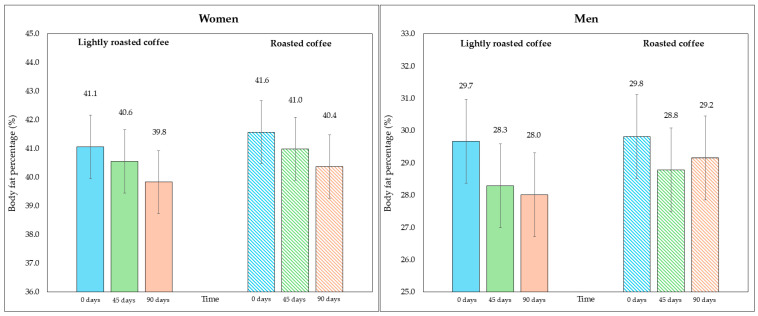
Changes in body fat percentage during the study, segmented by type of coffee (LRC is in solid color while RC is represented with a pattern of lines). The graph on the (**left**) represents the body fat percentage of women, while the graph on the (**right**) represents the values for men.

**Figure 3:**
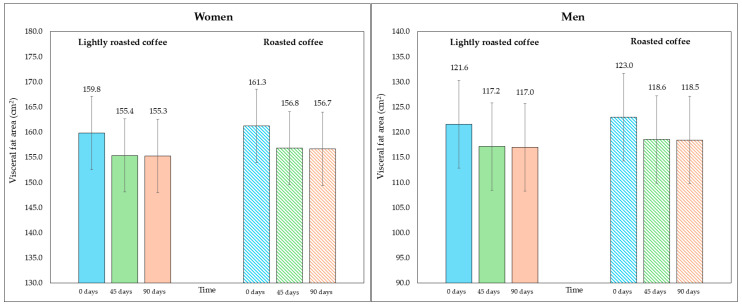
Changes in visceral fat area (in cm^2^) along the study, segmented by type of coffee (LRC is in solid color while RC is represented with a pattern of lines). The graph on the (**left**) represents the visceral fat area of women, while the graph on the (**right**) represents the values for men.

On the other hand, although the area of visceral fat did not decrease significantly at the end of the study, a trend was observed with the LRC remaining close to the limit of significance. This can be seen in the 95% CI (mean difference = −5.3 cm^2^, 95% CI, −9.6, −1.1; *p* = 0.058). It is possible that multiple contrasts (four) using the Holm adjustment, together with the loss of some values (three observations were lost due to errors in bioimpedance) could have affected this result. Figure 2 and Figure 3 show changes in body fat percentage and visceral fat area, segmented by type of coffee and by sex, due to the differences in the fat compartment between men and women. In Figures S2 and S3 (Supplementary Material), data are presented in graphs showing individual values as dots, which allows to see the interindividual variability in the study population.

**Figure 4:**
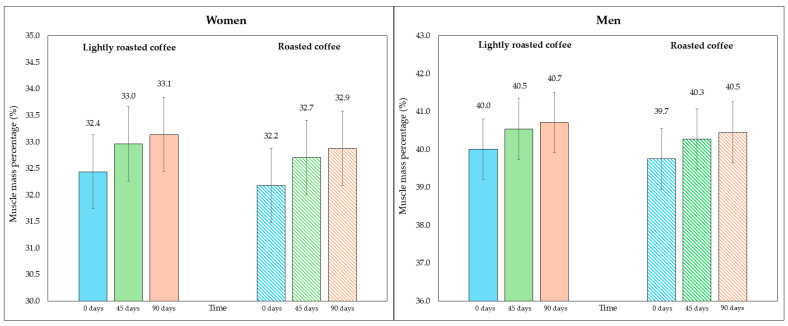
Changes in the percentage of skeletal muscle mass along the study, segmented by type of coffee (LRC is in solid color while RC is represented with a pattern of lines). The graph on the (**left**) represents the percentage of skeletal muscle mass of women, while the graph on the (**right**) represents the values for men.

Regarding skeletal muscle mass and its percentage, there was a modest, although significant, increase at the end of both interventions, as shown in Table 5. Figure 4 represents the average percentage of muscle mass in each intervention, segmented due to sex-related differences. In Figure S4 (Supplementary Material), data are displayed in a graph where individual values are represented by dots.

When comparing the final values after consuming both types of coffee, none of them showed a greater increase in skeletal muscle mass (mean difference = 0.3 kg, 95% CI, −0.06, 0.6; *p* = 0.213) nor in the increase in percentage of muscle mass (mean difference = 0.4%, 95% CI, −0.02, 0.8; *p* = 0.119).

Finally, there was no significant improvement after consumption of both types of coffee in any of the variables that make up the diagnostic criteria of the metabolic syndrome (Table 5). Systolic and diastolic blood pressure decreased at the end of both stages of the study, not reaching statistical significance. There also seemed to be a trend towards reducing fasting blood glucose in the RC intervention (mean difference = −2.7, 95% CI, −5.4, −0.08, *p* = 0.174).

## 4. Discussion

This crossover, randomized, and controlled study has been carried out in the context of an intervention that consisted of replacing volunteer’s habitual coffee with LRC or RC, following a realistic consumption rate in the Spanish population (three cups/day), without other lifestyle modifications such as reducing food intake or increasing physical activity.

After analyzing the participants’ diet, it was found that it did not meet the nutritional objectives for the Spanish population according to the Spanish Society of Community Nutrition [25]. As shown in Table 4, the caloric and lipid profile of the diet was unbalanced, with a greater energy contribution from fats and proteins to the detriment of carbohydrates. Saturated fat intake was higher than the recommended 10%. Dietary fiber intake was also inadequate, lower than that established in the nutritional objectives (Table 3 and Table 4). Moreover, participants had a sedentary behavior, according to the METs values (Table 2) that were ≤ 1.5 [26].

Although the volunteers had unbalanced diets, the results suggest that consuming three cups of coffee per day for 12 weeks could provide some benefits on body composition, improving body fat, and skeletal muscle mass ratios, without producing changes in body weight (Table 5).

These results agree with that described by other authors in similar intervention studies regarding the reduction in body fat percentage [21,27,28]. In contrast, in the present trial, no significant reduction in body weight or waist circumference was observed, although a trend was found regarding the reduction in visceral fat area after the consumption of LRC, which suggests a positive effect on abdominal obesity in these participants. The present results agree with another study with a similar approach (using two coffees with different degrees of roasting) as no reduction in weight, BMI, or waist circumference was found either [29].

These beneficial effects on the adipose tissue of overweight or obese people may be due to the hydroxycinnamic acids present in coffee, especially chlorogenic acid [12]. In this study, the consumption of LRC contributes to an intake of about 1200 mg per day of chlorogenic acid and its derivatives, while for RC it was approximately 450 mg, which would explain the superiority of LRC over RC and the tendency to reduce the visceral fat area. It should also be considered that there were no significant changes in terms of diet (Table 3 and Table 4) throughout the study and that participants’ physical activity, of a sedentary nature, did not change.

In studies carried out in murine models, it was observed that the administration of chlorogenic acid blocked the inflammation produced by a high-fat diet, inhibited the expression of peroxisome proliferator-activated receptor gamma (PPARγ) induced by the diet and the lipogenesis in the adipose tissue [30,31]. On the other hand, chlorogenic acid and other hydroxycinnamic acids present in coffee, such as ferulic acid, have been shown to exert inhibitory activity on cAMP phosphodiesterase [32]. This would result in an upregulation of adenosine monophosphate-activated protein kinase (AMPK), which would increase fatty acid oxidation. In addition, it could also inhibit pancreatic lipase, reducing the absorption of dietary lipids [32].

However, it must be considered that caffeine intake could also explain part of the results found in this study, since participants consumed about 70 mg per cup in the RC and 130 mg per cup in the LRC three times per day. In a meta-analysis of randomized controlled clinical trials, it was observed that caffeine intake could reduce body weight, BMI, and body fat in humans. The mechanisms by which caffeine could contribute to reducing fat weight come from an increase in energy expenditure, greater thermogenesis, and its antagonistic action on adenosine receptors, both in muscle mass and in the central nervous system, with a consequent increase in sympathetic activity [33].

On the other hand, this intervention has shown an increase in muscle mass and the percentage of muscle mass with the consumption of both coffees, which may explain the lack of significant changes in total body weight (Table 5). It is important to note that volunteers were instructed not to modify their habitual physical activity during the study, which was controlled by tracking daily activity levels with 24 h monitoring with accelerometers during one week in each intervention stage. This would rule out that the observed changes in the muscle compartment were due to increased exercise. There are few human studies analyzing the effects of coffee consumption on skeletal muscle mass. However, in an observational study, an association was found between higher coffee intake and a lower prevalence of low muscle mass [34]. The mechanisms by which these effects could occur are still being debated. In murine models of sarcopenia, coffee intake increased muscle mass, accelerated the regeneration of injured muscles and decreased some proinflammatory markers such as the interleukins IL-1α, IL-6, and tumor necrosis factor alpha (TNF-α) [35]. In another study in mice, it was observed that coffee consumption increased muscle hypertrophy through an increase in DNA synthesis of satellite cells, which participate in muscle growth and regeneration [36]. Furthermore, the expression of myostatin, a protein that limits muscle growth, was attenuated, and the regulation and phosphorylation of the Akt/PKB and mechanistic target of rapamycin (mTOR) pathways, known to regulate muscle protein synthesis, were increased [36]. Although it is unknown which compound would produce these effects on muscle mass, the authors discarded the idea that it was caffeine, since it has been shown to attenuate protein kinase B (Akt) phosphorylation in other studies [36]. Likewise, coffee consumption may improve insulin sensitivity and stimulate glucose uptake in muscle cells, which could potentially enhance skeletal muscle mass [37].

Regarding insulin sensitivity, some epidemiological studies have observed an association between greater coffee consumption and a lower incidence of type 2 diabetes mellitus [37]. In the study carried out by Sarriá et al. [21], it was shown that the intake of 510.6 mg of hydroxycinnamic acids and 121.2 mg of caffeine per day through the consumption of a green/roasted coffee blend reduced fasting glucose and insulin resistance. However, in the present study a significant reduction in blood glucose was not found, although after the consumption of RC a trend towards a reduction in fasting blood glucose was inferred, which was not significant due to Holm’s correction for multiple contrasts (Table 5). The higher caffeine intake with LRC compared to RC may have interfered with the beneficial effect of coffee polyphenols improving insulin sensitivity. In other studies, caffeine consumption has been shown to reduce insulin sensitivity in humans, although this effect may be masked by phenolic compounds producing a neutral effect on fasting blood glucose [37].

Additionally, there were no significant changes in systolic or diastolic blood pressure, although a slight decrease was observed at the end of the study with both coffees compared to the baseline level, despite the fact that caffeine intake was moderate–high (Table 5). Acute ingestion of high doses of caffeine (200–300 mg in a single dose) can raise blood pressure in individuals who are not habitual coffee drinkers [38]. However, prolonged consumption (2 weeks or more) does not appear to produce an increase in blood pressure compared to decaffeinated coffee [38]. In fact, habitual consumption of three to five cups of coffee seems to have a beneficial or neutral effect on blood pressure and the development of hypertension [39]. The presence of chlorogenic acid and its derivatives, as well as the potassium content of coffee, could partly explain the hypotensive effects of this food [27].

Finally, the consumption of both types of coffee was shown to have a neutral or slightly positive effect on HDL cholesterol levels and TG (Table 5). Although chlorogenic acid has been shown to decrease serum TG levels and increase HDL, it is possible that other components present in coffee such as diterpenes (mainly cafestol and kahweol), with hyperlipidemic effects, have masked the possible positive effects of the phenolic compounds in coffee [12,40,41].

### Limitations

Due to problems with the supply of the experimental coffee, the proportion of participants who started taking RC was higher than those who started with LRC, resulting in a participant ratio of 2:1 (two participants in the sequence RC-LRC: one in the LRC-RC sequence). As a result of some failures in the accelerometers, the physical activity of some volunteers could not be recorded. On the other hand, the few differences found between both coffees (LRC or RC) could be due to the fact that the content of chlorogenic acid and its derivatives, caffeine and other bioactive compounds in RC was high enough to produce a beneficial effect on body composition. This justifies the use of a higher dose of LRC compared to RC during the intervention, which might be considered a limitation. Ideally both groups should have consumed the same amount (in grams/day) of coffee; however, based on our team’s previous findings, where a green/roasted coffee blend providing 510.6 mg of hydroxycinnamic acids and 121.2 mg of caffeine per day was shown to positively affect blood pressure, blood lipids, and body weight in hypercholesterolemic subjects [14], we aimed to determine whether a higher dose of coffee-derived phenolic compounds could have a more pronounced effect in overweight or obese participants.

This was a free-living intervention to investigate the potential effect of two coffees with different phenolic content without further modification of other lifestyle factors such as diet or physical activity. We are aware that caloric restriction and increased energy expenditure through exercise would have probably added more impact on changes in body composition and metabolic syndrome markers. However, we selected this mild, non-intrusive study design as a more realistic approach that could be easily implemented into dietary habits by people with overweight/obesity, people in whom strict dietary regimes and increased physical activity usually have low adherence and little success in the long term. This approach implies that participants maintained their habitual diet, with differences in the food and caloric intake among them, and their physical activity was also controlled to minimize changes, which might have affected the results.

Finally, because of the complexity and duration of the study there was a high dropout rate (36.7%). In spite of this, the sample size was adequate to perform the statistical analysis; however, it was not high enough to perform a sub analysis and identify the participants who benefit most from the consumption of coffee rich in polyphenols.

## 5. Conclusions

Both coffees (the experimental and the control) were shown to significantly reduce body fat percentage and increase the percentage of muscle mass in overweight or obese people. In this sense, lightly roasted coffee seems to be superior in inducing positive changes in the adipose tissue.

Although, no changes in total body weight or variables related to metabolic syndrome were detected in the participants, moderate consumption of a coffee rich in polyphenol (three cups a day) could mitigate some of the negative effects on body composition of an unbalanced diet in overweight or obese people. The free-living nature of this intervention offers valuable insights into real-world applications.

However, the results reported here should be taken with caution, mostly due to the limited number of participants in the intervention study and the high interindividual variability observed. The present preliminary results will be completed with a larger sample size from this ongoing study. Importantly, the potential causes of the interindividual variability in the response to the intervention will be addressed by integrating the results on body composition and clinical biomarkers with genetic, metabolomic, and intestinal microbiota analyses along with other factors such as chronobiology, sleep quality, control of eating, etc. This comprehensive, multifactorial research approach should contribute to advancements in the knowledge of the causes of the different interindividual responses to the consumption of bioactive compounds, specifically a coffee rich in polyphenols and other bioactive substances.

## Figures and Tables

**Table 1:** Sociodemographic and socioeconomic characteristics of the participants involved in the intervention.

	Total *n* (%)	LRC-RC *n* (%)	RC-LRC *n* (%)	*p* Value
Women	23 (60.5%)	6 (60%)	17 (60.7%)	1.000
Men	15 (39.5%)	4 (40%)	11 (39.3%)	
Low educational level	2 (5.6%)	0 (0%)	2 (7.4%)	0.641
Medium educational level	6 (16.7%)	2 (22.2%)	4 (14.8%)	
High educational level	28 (77.8%)	7 (77.8%)	21 (77.8%)	
	Total	LRC-RC	RC-LRC	*p* value
Age (*n* = 38) ^a^	51.0 (9.8)	50.5 (11.8)	51.0 (7.3)	0.973
BMI (kg/m^2^) (*n* = 38)	30.2 ± 2.3	31.1 ± 1.6	29.9 ± 2.4	0.134
Monthly income per person (EUR) (*n* = 33) ^a^	1000 (900)	1350 (900)	925 (788)	0.363
Monthly income per family unit (EUR) (*n* = 34) ^a^	3000 (1800)	3000 (2000)	3300 (1500)	0.814

Descriptive data from categorical variables are expressed as the absolute frequency (*n*) and the relative frequency in percentage between parentheses. Superscript ^a^ indicates data that did not follow a normal distribution. Normally distributed data were expressed with the mean ± standard deviation while not normally distributed data were expressed with the median and the interquartile range in parenthesis. LRC-RC: participants allocated to lightly roasted coffee—roasted coffee; RC-LRC: participants allocated to roasted coffee—lightly roasted coffee. Some data are missing because participants declined to answer that question.

**Table 2:** Physical activity and energy expenditure of participants.

*n* = 37	Total
METs ^a^	1.11 (0.09)
Average daily kcals ^a^	442 (215)
Average hourly kcals ^a^	18.9 (10.1)
Average steps per day ^a^	7429 (3057)
REE (kcals/day)	1787 ± 313

METs: metabolic equivalents of task; REE: resting energy expenditure. All values are expressed in kcal/day or kcal per hour except METs, which are expressed as oxygen consumption [3.5 mL per kilogram of body weight per minute (mL/kg/min)]. The superscript ^a^ indicates that these variables did not follow a normal distribution; therefore, they are expressed as median and the interquartile range (in parenthesis). There was one volunteer from whom physical activity measurement could not be obtained, and another who could not have indirect calorimetry performed.

**Table 3:** Analysis of dietary intake throughout the study.

	Lightly Roasted Coffee			Roasted Coffee		
*n* = 38	Baseline (0 Days)	End (90 Days)	*p* Value	Baseline (0 Days)	End (90 Days)	*p* Value
Energy (kcal)	1972 ± 78	2039 ± 77	0.845	1985 ± 79	2092 ± 83	0.687
Proteins (g)	86.4 ± 3.6	85.8 ± 3.5	0.873	82.6 ± 3.6	91.9 ± 3.8	0.113
Carbohydrates (g)	185 ± 8.4	180 ± 8.3	0.544	168 ± 8.4	182 ± 8.8	0.275
Lipids (g)	88.2 ± 5.0	96.8 ± 5.0	0.259	100 ± 5.1	100 ± 5.3	0.996
SFA (g)	27.3 ± 1.7	28.1 ± 1.7	1.000	29.9 ± 1.7	30.2 ± 1.8	1.000
MUFA (g)	38.7 ± 2.4	41.7 ± 2.3	0.550	45.6 ± 2.4	44.3 ± 2.5	0.654
PUFA (g)	12.0 ± 1.4	15.2 ± 1.4	0.203	14.0 ± 1.4	14.5 ± 1.5	0.780
Alcohol (g)	6.9 ± 1.6	8.7 ± 1.6	0.812	5.5 ± 1.6	6.9 ± 1.7	0.812
Dietary fiber (g)	20.6 ± 1.6	22.3 ± 1.5	0.737	20.2 ± 1.6	23.1 ± 1.7	0.531

Data were analyzed with a linear mixed model. For each visit and variable analyzed, data are presented as marginal estimated means ± standard error of mean (SEM). *p* values were corrected using Holm’s method for multiple contrasts. *p* values showed in this table correspond to the contrasts between the first and the final visit of each intervention. A total of 17 observations were missing due to missing data. SFA: saturated fatty acids; MUFA: monounsaturated fatty acids; PUFA: polyunsaturated fatty acids.

**Table 4:** Contribution of macronutrients, alcohol, and fiber to the total energy intake of the volunteers.

	Lightly Roasted Coffee			Roasted Coffee		
*n* = 38	Baseline (0 Days)	End (90 Days)	*p* Value	Baseline (0 Days)	End (90 Days)	*p* Value
Proteins (%)	17.8 ± 0.6	17.3 ± 0.6	0.499	16.8 ± 0.6	17.8 ± 0.7	0.499
Carbohydrates (%)	37.6 ± 1.1	35.3 ± 1.1	0.114	34.2 ± 1.1	34.6 ± 1.1	0.765
Lipids (%)	39.9 ± 1.0	42.4 ± 1.0	0.107	44.9 ± 1.1	43.1 ± 1.1	0.180
SFA (%)	12.4 ± 0.4	12.2 ± 0.4	0.990	14.0 ± 0.4	13.0 ± 0.5	0.990
MUFA (%)	17.5 ± 0.7	18.6 ± 0.7	0.321	20.3 ± 0.7	19.1 ± 0.7	0.321
PUFA (%)	5.4 ± 0.4	6.3 ± 0.4	0.258	6.3 ± 0.4	6.2 ± 0.4	0.764
Alcohol (%)	2.5 ± 0.6	2.9 ± 0.6	1.000	2.0 ± 0.6	2.4 ± 0.6	1.000
Dietary fiber (%)	2.1 ± 0.1	2.2 ± 0.1	1.000	2.1 ± 0.1	2.2 ± 0.1	1.000

Data were analyzed with a linear mixed model. For each visit and variable analyzed, data are presented as estimated marginal means ± standard error of the mean (SEM). *p* values were corrected using Holm’s method. The *p* values shown in this table correspond to the contrasts between the first and last visits of each coffee intervention. A total of 17 observations were lost due to missing data. Data are expressed as the contribution of each macronutrient, alcohol, or fiber to the total energy of the diet in percentage. SFA: saturated fatty acids; MUFA: monounsaturated fatty acids; PUFA: polyunsaturated fatty acids.

**Table 5:** Changes in body weight, body composition, and components of metabolic syndrome.

	Lightly Roasted Coffee			Mean Difference (CI 95%)	*p* Value	Roasted Coffee			Mean Difference (CI 95%)	*p* Value
*n* = 38	First Visit t0 = 0 Days	Second Visit t2 = 45 Days	Final Visit t3 = 90 Days			First Visit t0 = 0 Days	Second Visit t2 = 45 Days	Final Visit t3 = 90 Days		
Body weight (kg)	85.5 ± 1.5	85.3 ± 1.5	85.6 ± 1.5	0.1 (−0.5, 0.7)	1.000	86.0 ± 1.5	85.8 ± 1.5	85.8 ± 1.5	−0.2 (−0.8, 0.4)	1.000
Fat mass (kg)	29.9 ± 1.0	29.2 ± 1.0	28.6 ± 1.0	−1.1 (−1.7, −0.5)	0.003 **	30.4 ± 1.0	29.7 ± 1.0	29.5 ± 1.0	−0.9 (−1.5, −0.2)	0.024 *
% of body fat	35.3 ± 0.9	34.5 ± 0.9	33.9 ± 0.9	−1.4 (−2.0, −0.8)	<0.001 ***	35.7 ± 0.9	34.9 ± 0.9	34.7 ± 0.9	−1.0 (−1.6, −0.4)	0.005 **
VFA (cm^2^)	140.7 ± 6.1	137.1 ± 6.1	135.3 ± 6.1	−5.3 (−9.6, −1.1)	0.058	142.1 ± 6.1	136.8 ± 6.1	138.4 ± 6.1	−3.8 (−8.0, 0.4)	0.218
Muscle mass (kg)	31.2 ± 0.7	31.5 ± 0.7	31.9 ± 0.7	0.7 (0.4, 1.0)	<0.001 ***	31.2 ± 0.7	31.6 ± 0.7	31.6 ± 0.7	0.4 (0.1, 0.8)	0.024 *
% of muscle mass	36.2 ± 0.6	36.7 ± 0.6	37.0 ± 0.6	0.8 (0.4, 1.1)	<0.001 ***	36.0 ± 0.6	36.6 ± 0.6	36.6 ± 0.6	0.7 (0.3, 1.0)	0.002 **
WC (cm)	96.5 ± 1.4	96.5 ± 1.4	96.7 ± 1.4	0.2 (−0.7, 1.1)	1.000	97.5 ± 1.4	97.6 ± 1.4	96.8 ± 1.4	−0.8 (−1.7, 0.1)	0.291
FBG (mg/dL)	92.5 ± 1.9	93.4 ± 1.9	93.0 ± 1.9	0.5 (−2.2, 3.1)	1.000	95.0 ± 1.9	98.6 ± 1.9	92.3 ± 1.9	−2.7 (−5.4, −0.08)	0.174
SBP (mmHg)	121.4 ± 2.8	122.0 ± 2.8	120.5 ± 2.8	−0.9 (−4.2, 2.4)	0.656	124.5 ± 2.8	124.5 ± 2.8	122.4 ± 2.8	−2.0 (−5.3, 1.2)	0.656
DBP (mmHg)	83.4 ± 1.7	82.8 ± 1.7	81.6 ± 1.7	−1.8 (−3.8, 0.1)	0.184	84.6 ± 1.7	85.0 ± 1.7	83.7 ± 1.7	−0.9 (−2.8, 1.1)	0.457
TG (mg/dL)	125.4 ± 10.3	131.0 ± 10.3	126.6 ± 10.3	1.2 (−13.0, 15.4)	1.000	133.6 ± 10.3	139.8 ± 10.3	127.4 ± 10.3	−6.3 (−20.5, 8.0)	1.000
HDL (mg/dL)	57.6 ± 2.6	60.5 ± 2.6	59.5 ± 2.6	1.9 (−0.5, 4.2)	0.340	59.9 ± 2.6	62.2 ± 2.6	58.2 ± 2.6	−1.7 (−4.1, 0.7)	0.340

Data were analyzed with a linear mixed model. For each visit and variable analyzed, data are presented as marginal estimated means ± standard error of the mean (SEM). *p* values were corrected using Holm’s method for the following contrasts: pre-post intervention, end of periods, and basal values for each intervention and type of coffee to analyze if there was a carry-over effect. Mean differences, confidence intervals (CI) and *p* values shown in this table correspond to the contrasts between the first and the final visit of each period (with LRC or RC). *p* < 0.05 are indicated with one asterisk (*), two asterisks if *p* < 0.01 (**) or three asterisks if *p <* 0.001 (***). WC: waist circumference; FBG: fasting blood glucose; SBP: systolic blood pressure; DBP: diastolic blood pressure; TG: triglycerides; HDL: high-density lipoprotein; VFA: visceral fat area.

## Data Availability

The original contributions presented in the study are included in the article/supplementary material, further inquiries can be directed to the corresponding author due to the data are part of an ongoing study.

## Supplementary Materials

The following supporting information can be downloaded at: https://www.mdpi.com/article/10.3390/nu16172848/s1. Figure S1. Flow diagram of the participants included in the GREENCOF study, based on the CONSORT 2010 (Consolidated Standards of Reporting Randomized Crossover Trials) guidelines. Figure S2. Changes in body fat percentage segmented by sex (women data are colored in red, while men in blue) and by type of coffee. Each dot represents an individual participant’s value. Figure S3. Changes in visceral fat area (in cm^2^) segmented by sex (women data are colored in red, while men in blue) and by type of coffee. Each dot represents an individual participant’s value. Figure S4. Changes in skeletal muscle mass percentage segmented by sex (women data are colored in red, while men in blue) and by type of coffee. Each dot represents an individual participant’s value. Table S1. CONSORT checklist of information to include when reporting randomized crossover trials.
