# Supplementary material for: Consumption of a Coffee Rich in Phenolic Compounds May Improve the Body Composition of People with Overweight or Obesity: Preliminary Insights from a Randomized, Controlled and Blind Crossover Study
Source: Nutrients. 2024 Aug 26;16(17):2848. doi: 10.3390/nu16172848 (PMC11397522; doi:10.3390/nu16172848)
Supplement: Supplementary file 1 [file nutrients-16-02848-s001.zip › nutrients-3151821-supplementary.pdf]

Article

Consumption of a coffee rich in phenolic compounds may improve the body composition of people with overweight or obesity: preliminary insights from a randomized, controlled and blind crossover study

Álvaro Fernández-Cardero [1], José Luis Sierra-Cinos [2,3], Laura Bravo [1] and Beatriz Sarriá [1,3*]

Supplementary information

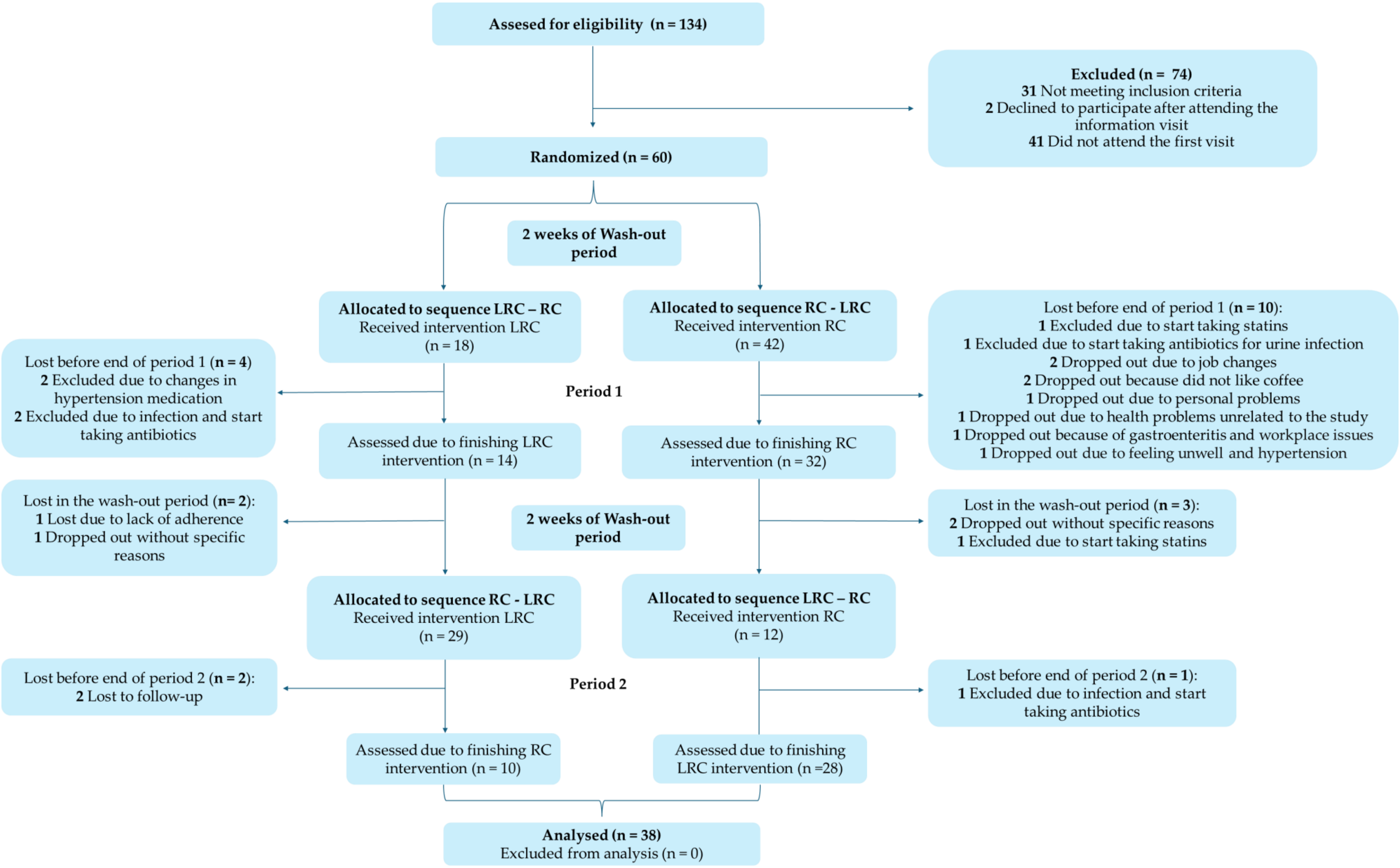

Figure S1. Flow diagram of the participants included in the GREENCOF study, based on the CONSORT 2010 (Consolidated Standards of Reporting Randomized Crossover Trials) guidelines.

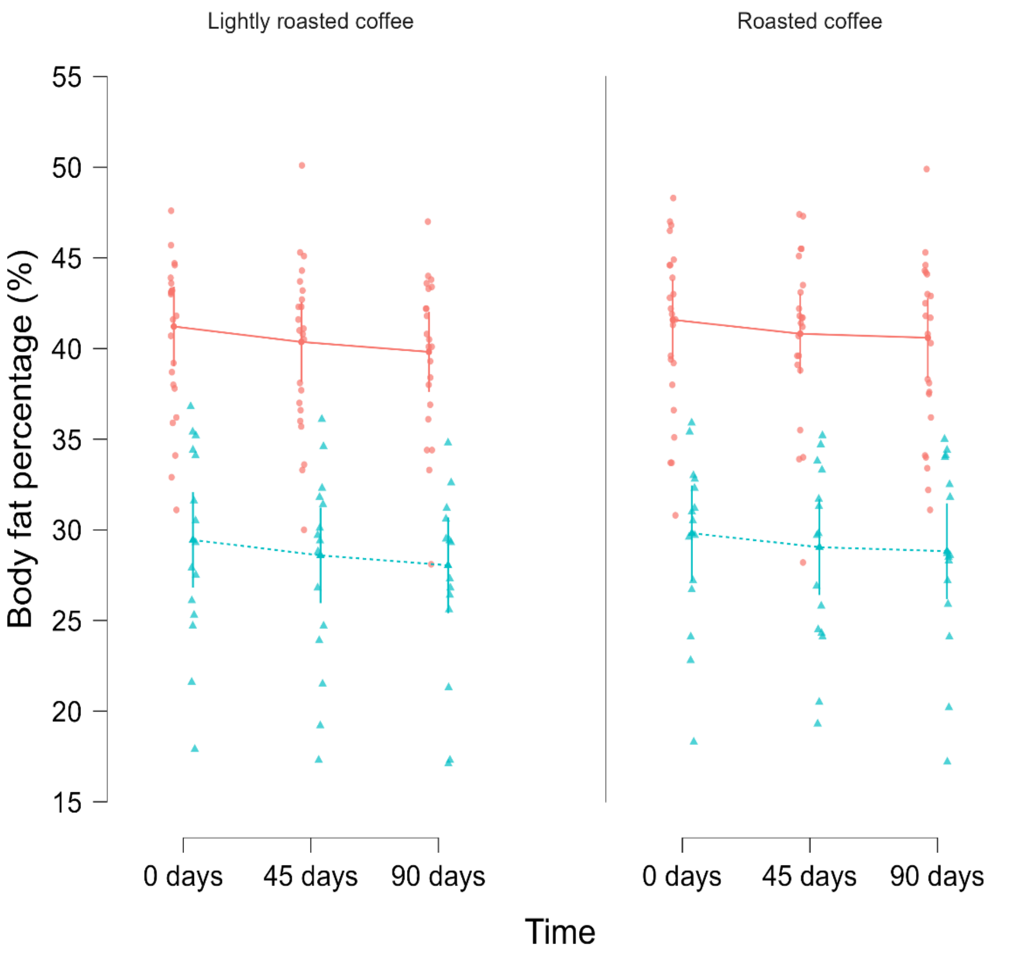

Figure S2. Changes in body fat percentage segmented by sex (women data are colored in **red**, while men in **blue**) and by type of coffee. Each dot represents an individual participant's value.

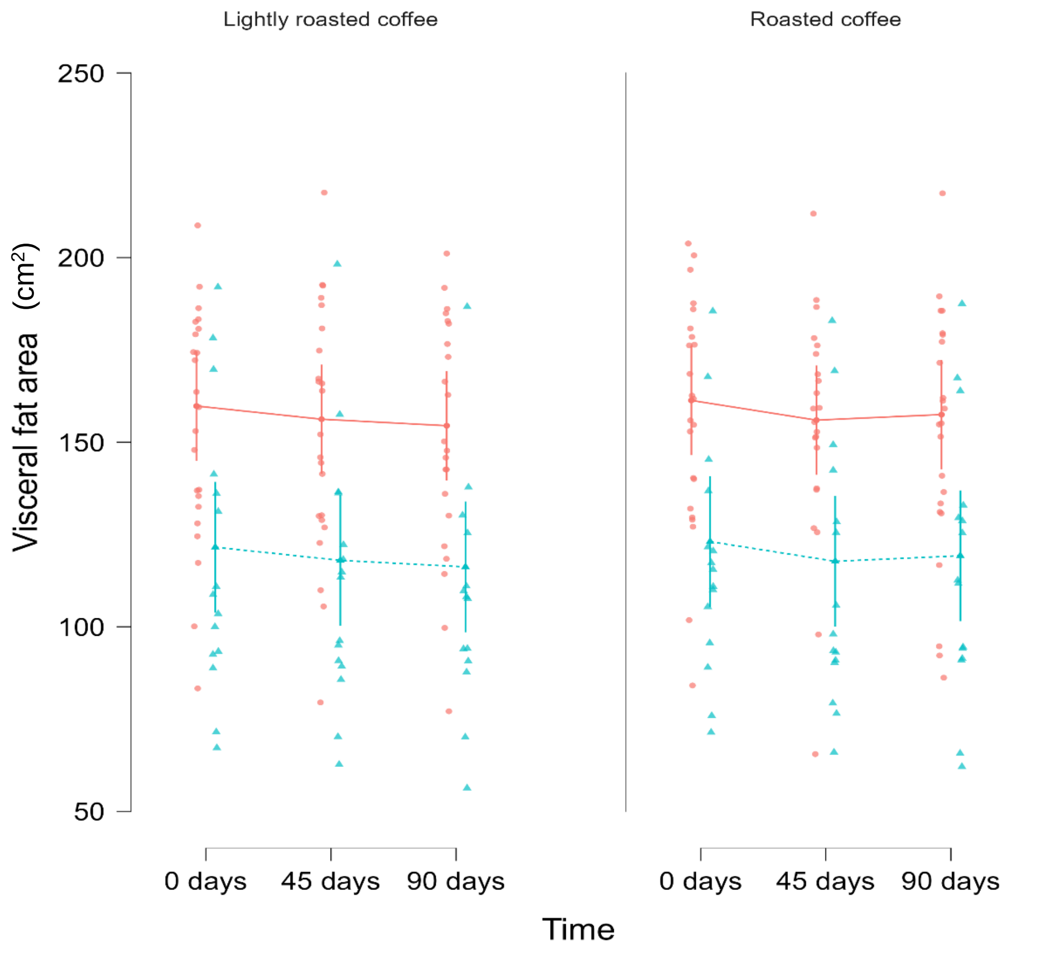

Figure S3. Changes in visceral fat area (in cm^2) segmented by sex (women data are colored in **red**, while men in **blue**) and by type of coffee. Each dot represents an individual participant's value.

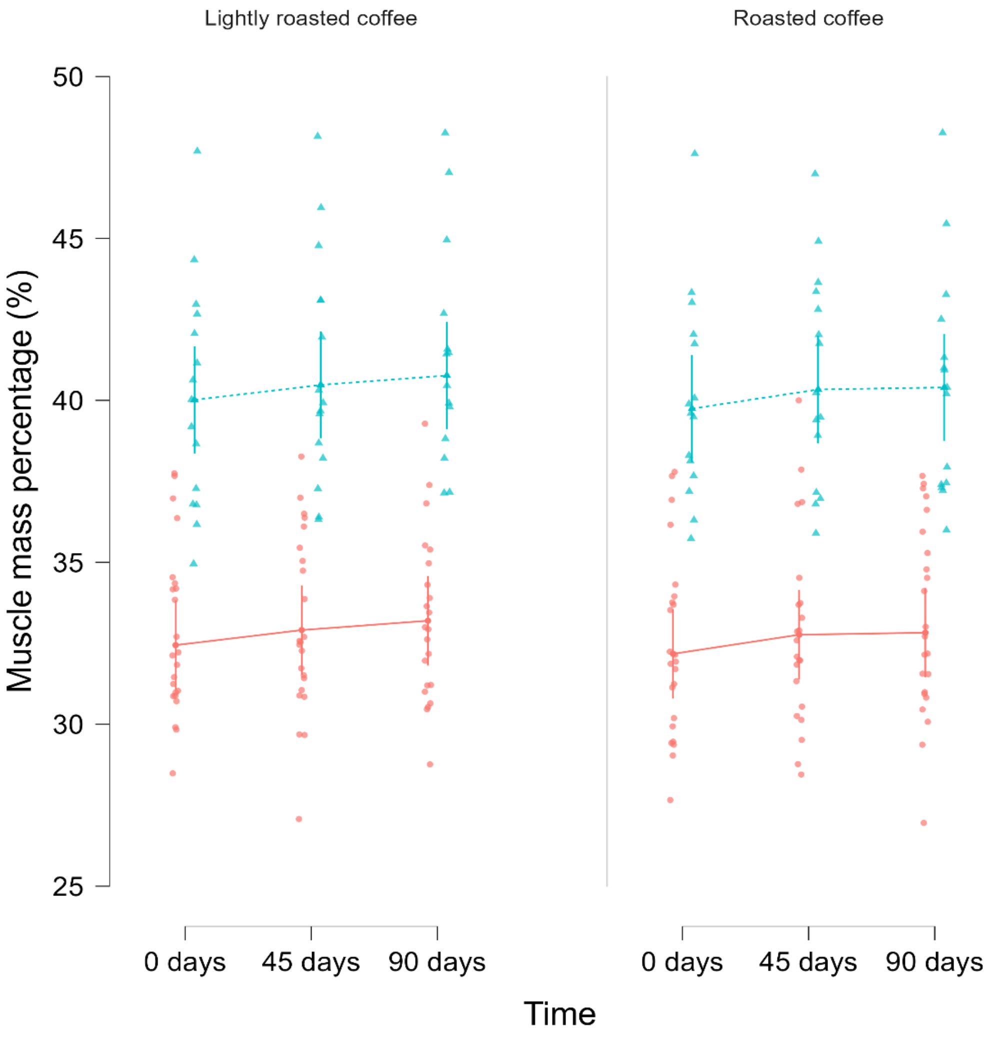

Figure S4. Changes in skeletal muscle mass percentage segmented by sex (women data are colored in **red**, while men in **blue**) and by type of coffee. Each dot represents an individual participant's value.

Table S1. CONSORT checklist of information to include when reporting randomized crossover trials.

Section/topic	Item Nº	Description	Reported on Page Number/Line Number	Reported on Section/Paragraph
Title†	1a	Identification as a randomized crossover trial in the title	Page 1; Line 1-5	Title
Abstract†	1b	Specify a crossover design	Page 1; Line 18-20	Abstract
**Introduction**				
Background‡	2a	Scientific background and explanation of rationale	Page 1-3; Line 35-99	Introduction
Objectives‡	2b	Specific objectives or hypotheses	Page 3: Line 100-107	End of Introduction
**Methods**				
Trial design†	3a	Rationale for a crossover design. Description of the design features including allocation ratio, especially the numberand duration of periods, duration of washout period, and consideration of carry over effect	Page 2-3; Line 110-127<br>Page 5; Line 197-198 (allocation)	Material and methods section (2), subsection 2.1. Study design and subsection 2.2. Inclusion /exclusion criteria, recruitment and randomization
Change from protocol‡	3b	Important changes to methods after trial commencement (such as eligibility criteria), with reasons	Page 15; Line 520-523 (change in allocation ratio)	Limitations section (4) subsection 4.1.
Participants‡	4a	Eligibility criteria for participants	Page 4-5; Line 169-187	Material and methods section (2), subsection 2.2. Inclusion /exclusion criteria, recruitment and randomization
Settings and location‡	4b	Settings and locations where the data were collected	Page 3; Line 121-124	Material and methods section (2), subsection 2.1. Study design
**Section/topic**	**Item Nº**	**Description**	**Reported on Page Number/Line**	**Reported on Section/Paragraph**

			**Number**	
Interventions†	5	The interventions with sufficient details to allow replication, including how and when they were actually administered	Page 3-4; Line 118-157<br><br>Page 5; Line 188	Material and methods section (2), subsections 2.1. Study design and<br>2.2. Inclusion /exclusion criteria, recruitment and randomization
Outcomes‡	6a	Completely defined prespecified primary and secondary outcome measures, including how and when they were assessed	Page 6; Line 265-275	Material and methods section (2), subsections<br>2.3.Diet control, measurement of energy expenditure and physical activity<br>2.4. Analysis of blood samples and blood pressure<br>2.5. Anthropometric and body composition measurements
Changes to outcomes‡	6b	Any changes to trial outcomes after the trial commenced, with reasons	Not applicable	Not applicable
Sample size†	7a	How sample size was determined, accounting for within participant variability	Page 6; Line 244-249	Material and methods section (2), subsection 2.6. Sample size calculation
Interim analyses and stopping guidelines‡	7b	When applicable, explanation of any interim analyses and stopping guidelines	Not applicable	Not applicable
**Section/topic**	**Item Nº**	**Description**	**Reported on Page Number/Line Number**	**Reported on Section/Paragraph**
**Randomization:**				

Sequence generation‡	8a	Method used to generate the random allocation sequence	Page 5; Line 198-200	Material and methods section (2), subsection 2.2. Inclusion /exclusion criteria, recruitment and randomization
Sequence generation‡	8b	Type of randomization; details of any restriction (such as blocking and block size)	Not applicable	Not applicable
Allocation concealment mechanism‡	9	Mechanism used to implement the random allocation sequence § (such as sequentially numbered containers),describing any steps taken to conceal the sequence until interventions were assigned	Not applicable	Not applicable
Implementation †	10	Who generated the random allocation sequence,§ who enrolled participants, and who assigned participants to thesequence of interventions	Page 5; Line 198-199	Material and methods section (2), subsection 2.2. Inclusion /exclusion criteria, recruitment and randomization
Blinding‡	11a	If done, who was blinded after assignment to interventions (for example, participants, care providers, those assessing outcomes) and how	Page 3-4; Line 131-132	Material and methods section (2), subsection 2.1. Study design
Similarity of interventions‡	11b	If relevant, description of the similarity of interventions	Page 15; Line 524-534	Section (4) Discussion, subsection 4.1. Limitations
Statistical methods†	12a	Statistical methods used to compare groups for primary and secondary outcomes which are appropriate for crossover design (that is, based on within participant comparison)	Page 6; Line 263-275	Material and methods section (2), subsection 2.7. Statistical analysis
**Section/topic**	**Item Nº**	**Description**	**Reported on Page Number/Line Number**	**Reported on Section/Paragraph**
Additional analyses‡	12b	Methods for additional analyses, such as subgroup analyses and adjusted analyses	Page 6; Line 263-275	Material and methods section (2), subsections 2.7. Statistical analysis
**Results**				

Participant flow (a diagram is strongly recommended) †	13a	The numbers of participants who were randomly assigned, received intended treatment, and were analysed for theprimary outcome, separately for each sequence and period	Page 6 and 7; Line 277-282; Figure S1	Results section (3) and supplementary material
Losses and exclusions†	13b	Nº of participants excluded at each stage, with reasons, separately for each sequence and period	Page 2 of supplementary material; Figure S1	Supplementary material
Recruitment‡	14a	Dates defining the periods of recruitment and follow-up	Page 5; Line 188	Material and methods section (2), subsection 2.2. Inclusion /exclusion criteria, recruitment and randomization
Trial end‡	14b	Why the trial ended or was stopped	Not applicable; Still going on	Not applicable; Still going on
Baseline data†	15	A table showing baseline demographic and clinical characteristics by sequence and period	Page 7; Line 290-295; Table 1	Results section (3)
Numbers analysed†	16	Number of participants (denominator) included in each analysis and whether the analysis was by original assignedgroups	Page 7; Line 290-295; Table 1	Results section (3)
**Section/topic**	**Item Nº**	**Description**	**Reported on Page Number/Line Number**	**Reported on Section/Paragraph**
Outcomes and estimation†	17a	For each primary and secondary outcome, results including estimated effect size and its precision (such as 95% confidence interval) should be based on within participant comparisons.¶ In addition, results for each intervention ineach period are recommended	Page 10; Line 362-367; Table 5	Results section (3)
Binary outcomes‡	17b	For binary outcomes, presentation of both absolute and relative effect sizes is recommended	Not applicable	Not applicable
Ancillary analyses‡	18	Results of any other analyses performed, including subgroup analyses and adjusted analyses, distinguishing prespecified from exploratory	Page 10; Line 362-367; Table 5	Results section (3)

Harms†	19	Describe all important harms or untended effects in a way that accounts for the design (for specific guidance, see CONSORT for harms)	Not applicable	Not applicable
**Discussion**				
Limitations†	20	Trial limitations, addressing sources of potential bias, imprecision, and if relevant, multiplicity of analyses. Consider potential carry over effects	Page 15; Line 521-550	Section (4) Discussion, subsection 4.1. Limitations
Generalizability ‡	21	Generalizability (external validity, applicability) of the trial findings	Page 15; Line 552-560	Section (5) Conclusion
Interpretation‡	22	Interpretation consistent with results, balancing benefits and harms, and considering other relevant evidence	Page 13, 14 and 15; Line 418-519	Section (4) Discussion
**Other information**				
Registration‡	23	Registration number and name of trial registry	Page 4; Line 158	Material and methods section (2), subsection 2.1. Study design
**Section/topic**	**Item Nº**	**Description**	**Reported on Page Number/Line Number**	**Reported on Section/Paragraph**
Protocol‡	24	Where the full trial protocol can be accessed, if available	Not available	Not available
Funding‡	25	Sources of funding and other support (such as supply of drugs), role of funders	Page 16; Line 581-583	Below the conclusions section (5), in Funding.

† Modified original CONSORT item. ‡ Unmodified CONSORT item. § Random sequence here refers to a list of random orders, typically generated through a computer program. This should not be confused with the sequence of interventions in a randomized crossover trial, for example receiving intervention A before B for an individual trial participant. ¶ A within participant comparison takes into account the correlation between measurements for each participant because they act as their own control, therefore measurements arenot independent. Article information: https://dx.doi.org/10.21037/jtd-23-1178
*As the checklist was provided upon initial submission, the page number/line number reported may be changed due to copyediting and may not be referable inthe published version. In this case, the section/paragraph may be used as an alternative reference.
